# Facial nerve schwannoma: A case report and review of the literature

**DOI:** 10.3892/ol.2014.2550

**Published:** 2014-09-18

**Authors:** MEI-CHIEN CHEN, TE-MING TSENG, SHIH-HAN HUNG, PO-YUEH CHEN

**Affiliations:** 1 Department of Otolaryngology Head and Neck Surgery, Shuang Ho Hospital, Taipei Medical University, Taipei 235, Taiwan, R.O.C.; 2 Department of Otolaryngology, Taipei Medical University Hospital, Taipei 110, Taiwan, R.O.C.; 3 Department of Otolaryngology, School of Medicine, Taipei Medical University, Taipei 110, Taiwan, R.O.C.

**Keywords:** facial nerve, schwannoma, facial palsy, hearing loss

## Abstract

A vestibular schwannoma, often termed an acoustic neuroma, is a type of benign primary intracranial tumor of the myelin-forming cells of the vestibulocochlear nerve. The typical clinical presentation often includes ipsilateral sensorineural hearing loss/deafness, vertigo and tinnitus. In the present study, the case of a young male patient who presented with recurrent unilateral facial palsy without hearing impairment is presented. The patient was diagnosed with vestibular schwannoma and received steroidal treatment with prednisolone for two weeks. The patient’s facial weakness recovered three weeks following treatment, however, the tumor subsequently grew. The patient then underwent Gamma Knife radiosurgery with a margin dose of 13 Gy. Six months after the radiosurgery, the tumor was stable without progression, and the patient’s facial nerve function and hearing remained intact.

## Introduction

Facial nerve schwannomas are rare benign tumors, which originate along the facial nerve. On imaging, those presenting as an enhancing cerebellopontine angle mass may be difficult to distinguish from vestibular schwannoma (also termed acoustic neuroma) and meningiomas. The geniculate ganglion is located in the temporal bone and contains cell bodies associated with facial nerve-specialized taste and general somatic sensory fibers. In the absence of pathological conditions, the geniculate ganglion is not usually observed under intravenous gadolinium-based enhancement.

Common clinical presentations of vestibular schwannoma include ipsilateral sensorineural hearing loss/deafness, disturbed sense of balance and altered gait, vertigo, nausea and vomiting, as well as a sensation of pressure in the ears (1). It is generally hypothesized that as the tumor increases in size, it compresses the brainstem and other cranial nerves. The facial nerves are rarely involved and, thus, facial paralysis is an uncommon symptom. However, Mackle *et al* (2) reported that 3.7% of vestibular schwannoma patients do not present with the typical audiovestibular symptoms that are associated with vestibular schwannoma. Written informed consent was obtained from the patient.

## Case report

In December 2012, a 36-year-old male was referred to Shuang Ho Hospital (Taipei, Taiwan) presenting with the sudden onset of right facial palsy three days previously. The patient had experienced right facial palsy two years previously and recovered completely following treatment at another hospital (Far Eastern Memorial Hospital, Taipei, Taiwan). The patient also experienced the sudden onset of right-sided tinnitus without noticeable hearing impairment or dizziness. On physical examination, a House-Brackmann (HB) grade III facial palsy was observed (3). No spontaneous or provoked nystagmus were identified during a head shaking maneuver. Furthermore, the tympanic membranes and nasopharynx were unremarkable.

**Figure 1:**
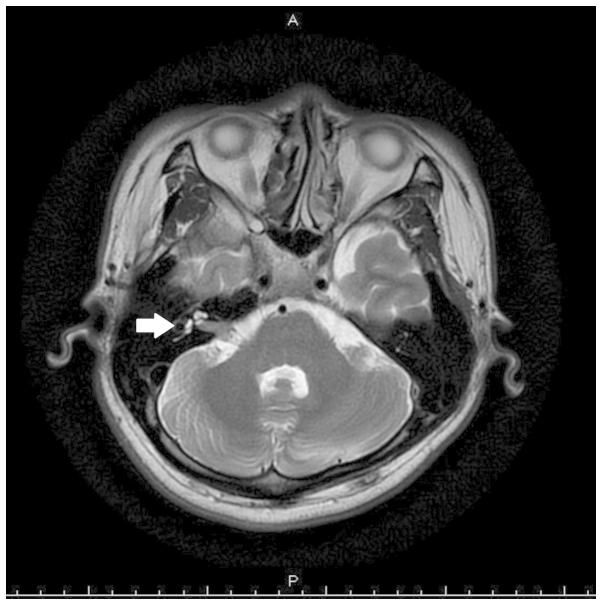
Contrast-enhanced T2-weighted magnetic resonance imaging reveals an intracanalicular tumor in the region of the cerebellopontine angle (shown by the arrow).

Pure tone audiometry did not reveal any hearing impairment and the patient’s speech recognition was 100% bilaterally. In addition, the auditory brainstem response appeared to be normal. Vestibular evoked myogenic potentials were symmetrical on either side. The caloric test revealed 38% right canal paresis, while facial electroneurography demonstrated 80% degeneration on the right side. The patient did not experience facial paresthesia or altered sensations of taste. Brain magnetic resonance imaging (MRI) demonstrated a mass (maximal diameter, 1.2 cm) in the right internal auditory canal (Fig. 1). No enhancement was observed at the labyrinthine portion of the facial nerve or at the geniculate ganglion.

Following steroidal therapy with prednisolone (1 mg/kg per day) for two weeks, the facial weakness improved to HB grade I. Considering the patient’s hearing status and that the tumor remained intracanalicular without any immediate risk of compressing other vital structures, conservative treatment (watchful waiting) with an MRI follow-up was performed.

**Figure 2:**
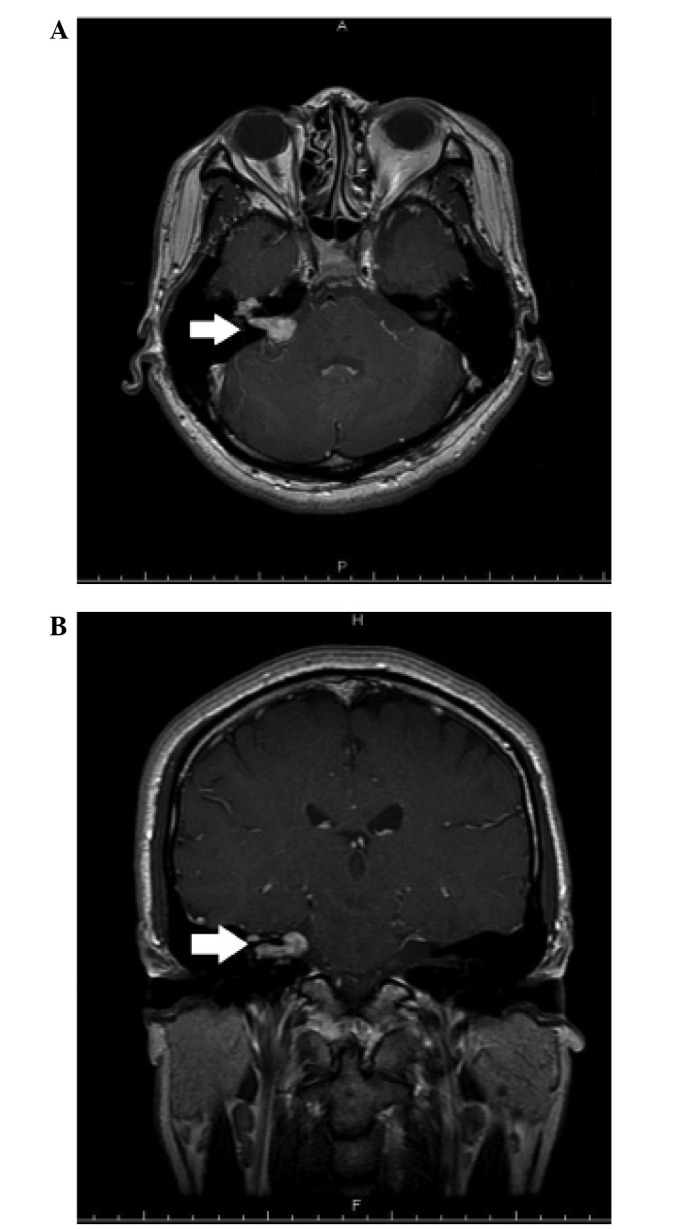
Contrast-enhanced T1-weighted magnetic resonance images. (A) Axial and (B) coronal views demonstrate an enhancing mass at the cerebellopontine angle and internal auditory canal, with involvement of the labyrinthine segment and geniculate ganglion (as shown by the arrows).

At the one-year follow-up the tumor had enlarged and extended along the facial nerve to the peri-geniculate area (Fig. 2). Right-sided tinnitus was observed, however, the patient’s hearing remained normal. Gamma Knife radiosurgery using a margin dose of 13 Gy was performed. Six months following radiosurgery, follow-up MRI imaging revealed that the tumor was stable without progression. In addition, the patient’s facial nerve function and hearing remained intact.

## Discussion

Facial nerve tumors are rare tumors of the temporal bone, which may involve any aspect of the facial nerve. However, facial nerve tumors predominantly present in the peri-geniculate area and the tympanic segment. Skip lesions or multiple segments of involvement are occasionally identified. Typical symptoms include the slow progression of facial nerve paresis or paralysis, as well as hearing loss, tinnitus, pain and vestibular symptoms. Furthermore, an ear canal mass may be present (9). Facial twitching followed by progressive paresis is also a common symptom of this type of tumor. Facial nerve tumors account for 5% of all cases of facial paralysis, worldwide and, therefore, must be considered in all cases of facial palsy (11).

Facial nerve schwannoma are the most common type of tumor involving the facial nerve. In a series analyzing 600 temporal bones, the incidence of intratemporal facial schwannoma was 0.8% (12). Perez *et al* (13) observed growth in four out of 13 facial schwannomas that were managed via expectant treatment, with a mean growth rate of 1.4 mm/year among the tumors that grew (although if all 13 tumors were considered, the mean growth rate was 0.42 mm/year). Facial nerve tumors are rarely considered during the differential diagnoses of enhancing cerebellopontine angle lesions. In the present case, the diagnosis was based on MRI enhancement of the geniculate ganglion, a structure that is located along the facial nerve within the temporal bone.

The management of facial nerve tumors has evolved from the performance of microsurgical excision with facial nerve repair (MER) to an increased adoption of more conservative techniques, which are regarded as facial nerve preservation approaches (14). These include observation with the watchful waiting approach, fallopian canal decompression and stereotactic radiosurgery. All of which are considered to be important treatment options for the management of facial nerve tumors (15). There is controversy regarding the resection of facial nerve tumors of any size when the facial nerve function is normal or near normal (HB grade I–II). In cases where tumors are determined to have a high probability of being facial nerve schwannoma, via imaging or electrophysiological analysis, preservation of the best possible facial nerve function must be prioritized regardless of the tumor size. An exception to this principle includes cases where the tumor is causing compressive symptoms, such as brainstem compression or labyrinthine erosion.

Stereotactic radiosurgery is an emerging treatment modality. Although it has been widely used for treating acoustic neuroma, there are few reports regarding its use in facial nerve tumors, however, the number is increasing (2). For facial nerve tumors, the advantages of stereotactic radiation include the avoidance of surgery, potential growth arrest of the tumor and possible preservation of facial nerve function. The selection of treatment is based on the intent to preserve facial nerve functions at the best possible level for the longest duration. If patients exhibit a progressively worsening clinical and radiological condition (HB grade II–IV and radiological evidence of growth or impending complications) treatments including, fallopian canal decompression, radiation or MER are considered to be treatment options that are dependent on the patient’s situation. Irradiation is an alternative for patients with early facial dysfunction (HB grade II–III) and a documented worsening of the clinical and radiological condition. It has a relatively small, although notable risk of worsening the auditory and facial function, which must be considered and discussed with the patient prior to determining the treatment plans (10).

The changes in facial function and tumor sizes of patients with facial nerve tumors, following stereotactic radiosurgery from all published studies identified during the literature search are presented in Table I. Approximately 4% of patients experienced a decline in facial nerve function and 7% exhibited tumor enlargement. The current treatment modality relies on observations with serial MRI for cases with no or limited facial nerve dysfunction.

The deterioration of facial nerve function or hearing, rapid tumor growth, or impending labyrinthine erosion or other complications require the consideration of conducting more aggressive treatment strategies. Irradiation or fallopian canal decompression of tumors is often employed for facial nerve function with an HB grade between II and IV. Microsurgical excision and facial nerve repair is generally delayed until the facial nerve function deteriorates to HB grades IV–VI. This is due to the consideration that postoperative facial nerve paralysis is expected to last for six to 18 months, which may be followed by an improvement to an HB grade III at best. In conclusion, the present case identified that enhancement of the geniculate ganglion is an important characteristic to identify when evaluating the MRI appearance of cerebellopontine angle masses. In addition, a more conservative treatment modality may allow a greater number of patients to experience improved long-term facial nerve function in the management of facial nerve tumors.

## Figures and Tables

**Table I:** Summary of variations in patient HB grade and tumor size following stereotactic radiation treatment for facial nerve tumors, obtained from the literature.

Study [year, (ref)]	Patients, n	Variation	

		HB grade	Tumor size
Litre *et al* [2007, (4)]	11	3 improved	4 decreased
		8 unchanged	6 stable
			1 increased
Kida *et al* [2007, (5)]	14	5 improved	8 decreased
		8 unchanged	6 stable
		1 worsened	
Nishioka *et al* [2009, (6)]	4	4 unchanged	2 decreased
			2 stable
Madhok *et al* [2009, (7)]	6	1 improved	3 decreased
		5 unchanged	3 stable
Hillman *et al* [2008, (8)]	2	1 improved	2 stable
		1 unchanged	
Wilkinson *et al* [2011, (9)]	6	1 improved	3 decrease
		5 unchanged	1 stable
			2 increased
Channer *et al* [2012, (10)]	3	1 improved	3 stable
		1 unchanged, 1 worsened	

HB, House-Brackmann.
